# Hypertensive diseases in pregnancy and subsequent lower risk of breast cancer: the common immune and antiangiogenic profile

**DOI:** 10.1038/bjc.2012.487

**Published:** 2012-10-23

**Authors:** L Carbillon

**Affiliations:** 1 Department of Obstetrics and Gynecology, Hôpital Jean Verdier, Assistance Publique-Hôpitaux de Paris, Paris 13 University, France

I read with great interest the study by Opdahl *et al* (2012) who reported from the links between two national registries including almost one million women having giving their first birth in Norway between 1967 and 2008, that women with hypertension or preeclampsia in their first pregnancy, as well as in any pregnancy, had a reduced breast cancer risk (hazard ratio (HR) 0.83, 95% confidence interval (CI) 0.77, 0.90 and HR 0.84, 95% CI 0.79, O.89, respectively). During follow-up, 15 856 women were diagnosed with invasive breast cancer in the registry, and these population-based data confirmed those of previous large case–control studies in reporting a reduced risk of breast cancer after hypertensive disorders of pregnancy in diverse states of America (Troisi *et al*, 1998; Innes and Byers, 2004; Terry *et al*, 2007). The Italian study by Talamini *et al*, (1997) and the Jerusalem Perinatal Study (Calderon-Margalit *et al*, 2009) were not specifically designed to address this particular link and were based on the inclusion of only 28 and 63 breast cancer cases, respectively, their conflicting conclusions possibly reflecting a particular genetic susceptibility or misclassification of patients with pregnancy-induced hypertension without diagnosis of preeclampsia.

In their study Opdahl *et al* (2012) discussed the possible role of confounding factors in this now well-assessed but intriguing association, indicating that obesity and maternal metabolic factors may have an important role in both diseases. However, the available recent literature suggests another approach for investigating these links. Indeed, the overrisk of nulliparity (HR 1.26, CI 1.12, 1.42) and consequently the protection effect of parity against breast cancer, was observed by the same group of researchers in women from three Norwegian counties who were born between 1886 and 1928 (Opdahl *et al*, 2011). This study confirmed fully other previous data from a meta-analysis of eight studies from the Nordic countries establishing that nulliparity was associated with a remarkably similar 30% increase in breast cancer risk compared with parous women (Ewertz *et al*, 1990).

Furthermore, a number of recent studies indicate that biological features reported in preeclampsia are not unique to this disorder, existing in normal pregnancies at a lower level (Redman and Sargent, 2005; Palm *et al*, 2011). Low-grade maternal inflammation is present in all pregnancies and is merely more pronounced in preeclampsia (Redman and Sargent, 2005). An excessive inflammatory response, cytokines and complement activation (Redman and Sargent, 2005) as part of a T-helper cell (Th1)-like inflammatory reaction at the expense of Th2 phenomena, is a common pathway to preeclampsia (Boij *et al*, 2012). Vascular endothelial growth factor inhibitors like soluble vascular endothelial growth factor receptor-1 (sVEGFR-1 or sFlt1) are produced by the placenta with a progressive increase from mid- to late gestation in normal pregnancies (Palm *et al*, 2011), but placental hypoxia (possibly in synergy with excessive inflammatory reaction) stimulates much earlier and excessive production of sFlt1, which binds and deactivates circulating VEGF (Redman and Sargent, 2009), leading to the final clinical emergence of preeclampsia in a two-stage model. Troisi *et al* (2008) also showed that maternal circulating angiogenic factors measured at delivery reflected maternal blood pressure increases from mid- to late pregnancy in uncomplicated pregnancies, as well as in hypertensive pregnancies, and that both balance in antiangiogenic/angiogenic circulating factors and blood pressure variations in pregnancy was a continuum from normal range to preeclampsia.

Strikingly, a Th1-like inflammatory reaction and antiangiogenic profile is similarly associated to a breast cancer protection or better prognosis. Using an integrated molecular approach of breast cancer by modelling and data integration on genomic models, Kristensen *et al* (2012) recently demonstrated that the strongest predictor of good outcome in breast cancer was the acquisition of a ‘gene signature’ that favored a high Th1/cytotoxic T-lymphocyte response at the expense of Th2-driven humoral immunity. Significantly higher levels of PlGF and VEGF were found in tumour tissue (Maae *et al*, 2012), and associated with recurrence, metastasis and patient mortality (Parr *et al*, 2005), while the promising therapy with the targeted monoclonal antibody bevacizumab acts as a anti-VEGF agent (Wu *et al*, 2006; Nielsen *et al*, 2010; Bear *et al*, 2012) (with possible severe adverse effects among which not the least common is indeed hypertension).

Thus, a body of evidence supports that the Th1-like inflammatory and antiangiogenic profile observed in hypertensive or preeclamptic patients could be a marker for a better immune and anti-angiogenic response to subsequent breast lesions in women with hypertensive disorders during pregnancy, and at least in part underlie the negative correlation between the two conditions. A thorough exploration of these issues could strongly help develop innovative approaches for prevention and treatment of breast cancer.
